# Evaluation of phenolic profile, antioxidant and anticancer potential of two main representants of Zingiberaceae family against B164A5 murine melanoma cells

**DOI:** 10.1186/0717-6287-48-1

**Published:** 2015-01-12

**Authors:** Corina Danciu, Lavinia Vlaia, Florinela Fetea, Monica Hancianu, Dorina E Coricovac, Sorina A Ciurlea, Codruţa M Şoica, Iosif Marincu, Vicentiu Vlaia, Cristina A Dehelean, Cristina Trandafirescu

**Affiliations:** Department of Pharmacognosy, Faculty of Pharmacy, University of Medicine and Pharmacy Victor Babes”, Eftimie Murgu Square, No. 2, Timisoara, 300041 Romania; Department of Pharmaceutical Technology, Faculty of Pharmacy, University of Medicine and Pharmacy Victor Babes”, Eftimie Murgu Square, No. 2, Timisoara, 300041 Romania; Department of Chemistry and Biochemistry, University of Agricultural Sciences and Veterinary Medicine of Cluj-Napoca, Mănăştur Str.,No. 3-5, Cluj-Napoca, 400372 Romania; Department of Pharmacognosy, Faculty of Pharmacy, University of Medicine and Pharmacy “Gr.T.Popa”, Iasi, Romania; Department of Toxicology, Faculty of Pharmacy, University of Medicine and Pharmacy “Victor Babes”, Eftimie Murgu Square, No. 2, Timisoara, 300041 Romania; Department of Pharmaceutical Chemistry, Faculty of Pharmacy, University of Medicine and Pharmacy “Victor Babes”, Eftimie Murgu Square, No. 2, Timisoara, 300041 Romania; Faculty of Medicine, University of Medicine and Pharmacy “Victor Babes”, Eftimie Murgu Square, No. 2, Timisoara, 300041 Romania; Department of Organic Chemistry, Faculty of Pharmacy, University of Medicine and Pharmacy “Victor Babes”, Eftimie Murgu Square, No. 2, Timisoara, 300041 Romania

**Keywords:** *Curcuma longa* Linnaeus, *Zingiber officinale* Roscoe, Polyphenols, Antioxidant, Melanoma

## Abstract

**Background:**

*Curcuma longa* Linnaeus and *Zingiber officinale* Roscoe are two main representatives of *Zingiberaceae* family studied for a wide range of therapeutic properties*,* including: antioxidant, anti-inflammatory, anti-angiogenic, antibacterial, analgesic, immunomodulatory, proapoptotic, anti-human immunodeficiency virus properties and anticancer effects. This study was aimed to analyse the ethanolic extracts of *Curcuma rhizome* (*Curcuma longa* Linnaeus) and *Zingiber rhizome* (*Zingiber officinale* Roscoe) in terms of polyphenols, antioxidant activity and anti-melanoma potential employing the B164A5 murine melanoma cell line.

**Results:**

In order to evaluate the total content of polyphenols we used Folin-Ciocâlteu method. The antioxidant activity of the two ethanolic extracts was determined by DPPH assay, and for the control of antiproliferative effect it was used MTT proliferation assay, DAPI staining and Annexin-FITC-7AAD double staining test. Results showed increased polyphenols amount and antioxidant activity for *Curcuma rhizome* ethanolic extract. Moreover, 100 μg/ml of ethanolic plant extract from both vegetal products presented in a different manner an antiproliferative, respectively a proapoptotic effect on the selected cell line.

**Conclusions:**

The study concludes that *Curcuma rhizome* may be a promising natural source for active compounds against malignant melanoma.

## Background

For centuries, plants, plant products or pure active phytocompounds have been successfully used for the benefits of human health. *Zingiberaceae* family also known as ginger family comprises a number of approximately 52 genera and over 1300 species of aromatic plants [1]. Among this high number of representatives some species have been reported for their therapeutic properties both in classical and ethno medicine [2]. *Curcuma longa* Linnaeus and *Zingiber officinale* Roscoe are two main representatives of *Zingiberaceae* family studied for a wide range of therapeutic properties. *Curcuma longa* Linnaeus, popular name-turmeric, is an aromatic, nutraceutical plant. The vegetal product of this plant, the root, have been intensively used, under different pharmaceutical formulations in Indian traditional medicine (Ayurveda) for different ailments, namely for wounds, acne, parasitic infection (local administration) and common cold, urinary tract disease and liver disease (systemic administration) [3]. Numerous experimental studies regarding the therapeutic activity of turmeric reported a plethora of pharmacological properties of this vegetal extract, including: antioxidant, anti-inflammatory, anti-angiogenic, antibacterial, analgesic, immunomodulatory, proapoptotic, anti-human immunodeficiency virus properties, being also studied in arthritis, diabetes, Alzheimer’s disease [4–8]. The major active compound responsible for the pharmacodynamic action is the polyphenol curcumin [9, 10]. Additionally, this natural polyphenol has been described as an anticancer agent, both *in vitro* and *in vivo* on a wide range of cancer types, such as colon, pancreatic, liver, cervical, pulmonary, thymic, brain, breast and bone cancer [11–13]. Recent studies intensively support the role of polyphenols in the prevention of degenerative diseases, like cardiovascular affections and cancers. Different fruits, vegetables, cereals, olive oil, chocolate and beverages, such as green tea, and red wine represent main sources of natural polyphenols [14, 15].

Together with turmeric, another exceedingly studied nutraceutical aromatic plant from *Zingiberaceae* family is *Zingiber officinale* Roscoe. Different types of extract from the root of ginger have been used in Ayurvedic and Chinese traditional herbal medicine in order to treat indigestion, vomiting, arthritis, rheumatism, pains, cramps, fever and infection [16]. The main pharmacological actions of active compounds extracted from ginger root reported by *in vitro* and *in vivo* test attributed to its active phytocompounds were: anti-inflammatory, antioxidant, antiemetic, anticancer, anticoagulant, immunomodulatory, antihyperglycemic, hypolipidemic, analgesic, and cardioprotective properties [6, 16–19]. The main phytochemical constituents of the root, the vegetal product of this plant, responsible for the therapeutic action are gingerols, shogaols, paradols, gingerdiols, and zingerone [6, 20]. Regarding its anticancer properties, recent studies have indicated a beneficial effect in case of liver, endometrial, ovarian and prostate cancer [21–24]. Furthermore, ginger was described as an anti-emetic agent in cancer chemotherapy [25]. Ginger was also reported to reduce the side effects of doxorubicin and cisplatin [26].

Skin cancers include basal cell carcinoma, squamous cell carcinoma and malignant melanoma. The first two types of skin cancer are the most frequent malignant neoplasms among fair-skinned population [27]. Recent studies report that in the last five decades the incidence of melanoma was also increasing especially in the case of white population [28]. Albeit malignant melanoma is less frequent than the other two types of skin cancer, is the most dangerous. It is responsible for most deaths due to its highly metastatic potential and resistance to chemotherapy [29]. One of the latest studies regarding the incidence in Europe shows that the highest rates are recorded in Nordic countries, especially in Switzerland, while Grece and other Mediterranean countries are at the opposite pole [30].

The aim of this study was to determine the total polyphenol content and the antioxidant activity of two ethanolic extracts obtained from the above mentioned species, and to perform preliminary *in vitro* tests regarding a possible antiproliferative and/or proapoptotic effect on murine melanoma cell line B164A5.

## Results

### Polyphenols content of the two ethanolic extracts

**Figure 1:**
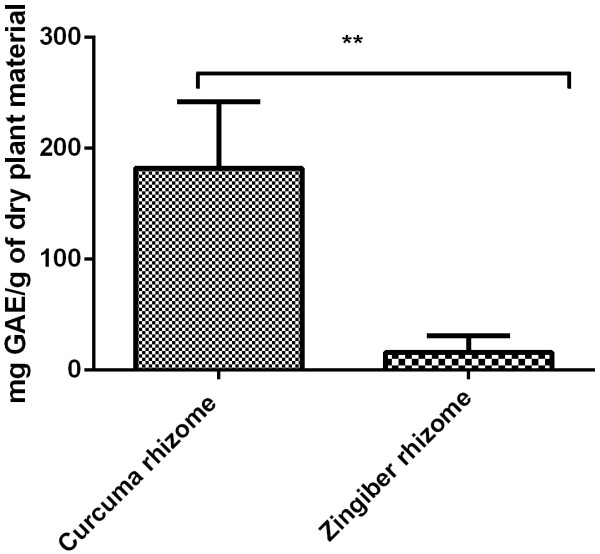
**Total pholiphenol content (mg P/1 ml extract) as revealed by Folin-Ciocalteu assay for** ***Curcuma rhizome*** **and** ***Zingiber rhizome.***

In the case of ethanolic extract obtained from *Curcuma rhizome*, the amount of polyphenols was 182 ± 0.6 mg GAE/g of dry plant material. A statistically significant decreased value was noticed in case of *Zingiber rhizome* ethanolic extract, namely 16 ± 0.15 mg GAE/g of dry plant material (Figure 1). Unpaired Student *t* test was used to determine the statistical difference between the two groups, the results were statistically significant (p value = 0.0097).

### Antioxidant capacity

**Figure 2:**
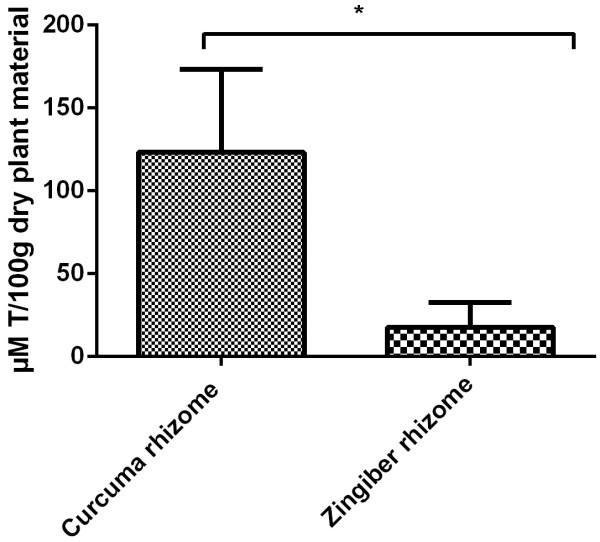
**Antioxidant capacity (mM T/1 ml extract) as revealed by DPPH assay for** ***Curcuma rhizome*** **and** ***Zingiber rhizome.***

The investigation continued with the analysis of the antioxidant capacity of the two extracts. In this regard it was applied an assay based on the measurement of the reducing ability of antioxidants toward DPPH^**.**^. Our results showed an increased antioxidant capacity for *Curcuma rhizome* ethanolic extract, 123.2 ± 5 μM T/100 g dry weight (dw) extract as compared to *Zingiber rhizome* ethanolic extract where a value of 17.7 ± 1.5 μM T/100 g dry weight (dw) (Figure 2). Unpaired Student *t* test was used to determine the statistical difference between the two groups, the results were not statistically significant (p value = 0.0249).

### Antiproliferative activity of the two ethanolic extracts

**Figure 3:**
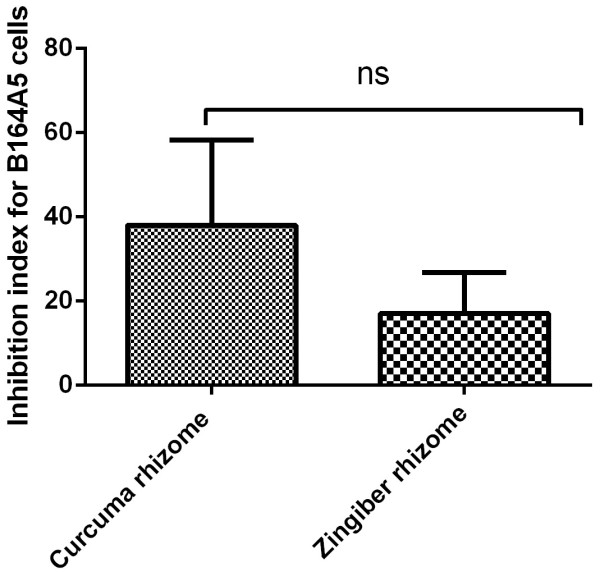
**Inhibition index for B164A5 cells as revealed by MTT assay after 48 h incubaton with 100 μg/ml ethanol extract of** ***Curcuma rhizome*** **and** ***Zingiber rhizome.***

In order to observe a possible antiproliferative or even cytotoxic effect of the two analyzed samples on murine melanoma B164A5 cell line, MTT proliferation assay was conducted as described in the Material and Methods section. 100 μg/ml of plant ethanol extracts showed after a period of incubation of 48 h an inhibition index of 38 ± 35 for *Curcuma rhizome* extract while in case of *Zingiber rhizome* extract the inhibition index was 17 ± 16 (Figure 3). Unpaired Student *t* test was used to determine the statistical difference between the two groups, the results were not statistically significant (p value = 0.4028). Preliminary studies employing lower concentrations of extracts were performed but the inhibition index was to low in order to be depicted.

**Figure 4:**
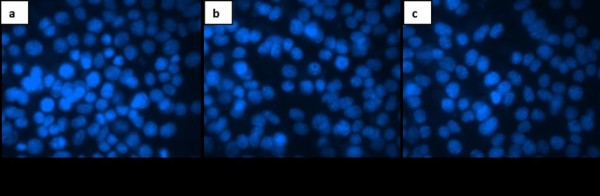
**DAPI staining after 48 h incubaton of B164A5 cells with a) Medium; b) 100 μg/ml ethanol extract of** ***Curcuma rhizome*** **; c) 100 μg/ml ethanol extract of** ***Zingiber rhizome.***

In addition, we decided to verify the apoptosis induced by the two ethanolic extracts on B164A5 cells using DAPI staining.After staining the cells with this reagent, the nucleus observed under fluorescent microscope was colored in blue. After 48 h incubation with the two extracts a number of cells presented nuclear fragmentation, condensed chromatin filaments or nuclear condensation as a sign of loss of cell membrane integrity. These characteristics were observed more frequently in case of the cells incubated with *Curcume rhizome* extract as compared to the ones incubated with *Zingiber rhizome* extract (Figure 4). Preliminary studies employing lower concentrations of extracts were performed but no or decreased signs of apoptosis could be detected.

**Figure 5:**
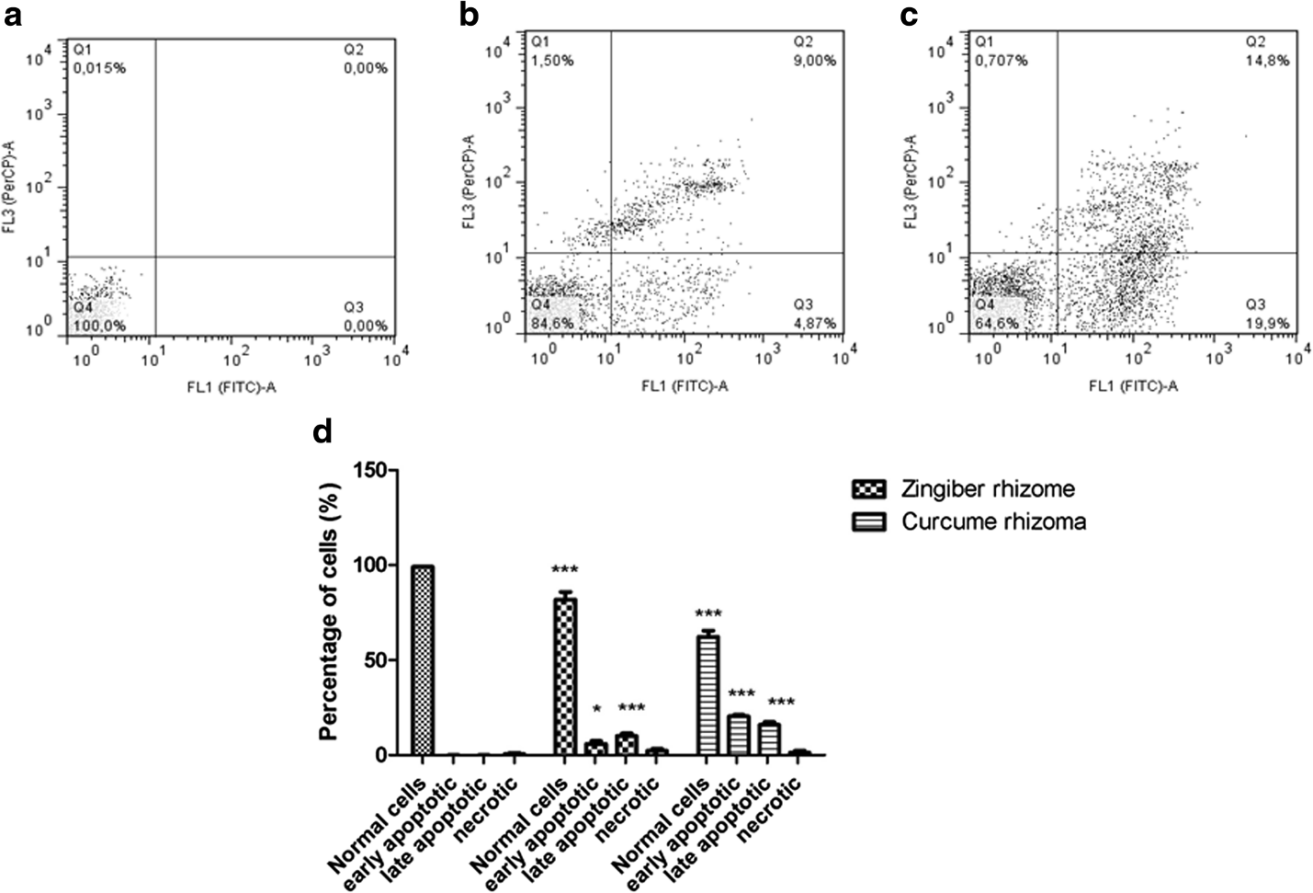
**Annexin-FITC-7AAD double staining after 48 h incubaton of B164A5 cells with: a) Medium; b) 100 μg/ml ethanol extract of** ***Curcuma rhizome*** **; c) 100 μg/ml ethanol extract of** ***Zingiber rhizome*** **; d) The ensemble image together with statistical analyze of a,b,c.**

In order to observe the apoptotic events (early apoptosis and late apoptosis) Annexin-FITC-7AAD double staining was performed. Results showed after an incubation period of 48 h a percentage of 5.935 ± 1.5 early apoptotic cells in case of *Zingiber rhizome* extract and 20.45 ± 0.77 early apoptotic cells in case of *Curcuma rhizome* extract. For late apoptotic cells the percentage was 10 ± 1.4 in case of *Zingiber rhizome* extract and 15.9 ± 1.55 in case of *Curcuma rhizome* extract (Figure 5). These results were statistically significant as revealed by Two-Way ANOVA followed by Bonferroni post test. Preliminary studies employing lower concentrations of extracts were performed but no or decreased signs of apoptosis could be detected.

## Discussions

It is well known that natural polyphenols act as antioxidants therefore a diet rich in fruits, vegetables, cereals, extra virgin olive oil, red wine and tea (Mediterranean diet) would be of real practical interest in order to counteract some important pathologies like cardiovascular disease, some types of cancer, Alzheimer’s disease, tooth decay or different infections [15, 31, 32]. Polyphenols are plant secondary metabolites and *Curcuma rhizome* and *Zingiber rhizome* have been reported as vegetal products that contain this type of phytochemicals [33–36]. It is well known that the amount of active agents is widely varying depending on the extraction conditions (solvent, temperature, extraction time) [37].

The aim of the present study was to analyze a possible anticancer potential of the two ethanolic extracts of curcuma and ginger root against B164A5 murine melanoma cell line. Extraction was performed by using ethanol as a solvent, solid: liquid ratio 1:50 at 70°C for 2 h. The extraction conditions were chosen based on a previous study conducted by the group of Surojanametakul *et al.*, who screened a wide range of parameters including solvent type, ratio and temperature, and have showed best results in terms of amount of extracted polyphenols [38].

In order to determine the amount of polyphenols we have chosen the Folin Ciocalteu assay. The procedure is convenient, simple, and reproducible [39]. Using other conditions not targeted to these two vegetal products, in a large screening study of 32 selected herbs, the group of Wojdyło *et al.*, detected 172 ± 0.12 mg GAE/g of dry plant material for *Curcuma rhizoma*[40]. In the same conditions as the ones we have used the group of Surojanametakul *et al.*, obtained for *Curcuma rhizoma* 146.65 mg GAE/g of dry plant material [38]. Other values for *Curcuma rhizoma* polyphenols were reported by the group of Mongkolsilp *et al.*, namely 122 mg GAE/g of dry plant material [41]. For two varieties of root of Malaysian *Zingiber officinale*, namely *Halia Bentong* and *Halia Bara*, the group of Ghasemzadeh *et al.*, recorded 10.22 ± 0.87 (*Halia Bentong*) and 13.5 ± 2.26 (*Halia Bara*) polyphenols expressed as mg gallic acid/g of dry plant material [42]. The group of Otunola *et al.*, reported a value of 22.09 total polyphenols expressed as mg gallic acid/g of dry plant material for ginger root [43].

Further investigations were conducted in order to determine the antioxidant capacity. We have measured the radical-scavenging activity of antioxidants against the free radical 1,1-diphenyl-2-picrylhydrazyl (DPPH). DPPH is a very common assay used to test the antioxidant capacity of vegetal extracts [44]. Results showed an increased antioxidant capacity for *Curcuma rhizome* ethanolic extract, as compared to *Zingiber rhizome* ethanolic extract. It can be observed a direct correlation between the amount of polyphenols detected in the selected vegetal products and the antioxidant capacity. Polyphenols have been intensively investigated for their antioxidant properties. It seems that the mechanism involves the modulation of oxidative stress [14].

To reach the final aim of our study we have screen for an antiproliferative, respectively proapoptotic effect of the two ethanolic extracts on the murine melanoma B164A5 cell line. As explained in the results section MTT proliferation assay revealed that at a dose of 100 μg/ml the extracts present antiproliferative capacity. Several reports regarding the antiproliferative activity of the two ethanolic extracts have been described in the literature, but none targeted towards B164A5 murinic melanoma cells.

Aqueous turmenic extracts were found to inhibit the proliferation of cultured bovine smooth muscle cells [45]. *Curcuma longa* extract was also found to inhibit the proliferation of rat’s hepatic stellate cells [46]. Additionally it was reported that the extract has cytotoxic effect with different IC_50s_ in breast and lung cancer cell lines [47, 48]. Cytotoxicity was also observed in case of lymphocytes and Dalton’s lymphoma [49]. Curcumin was found to sensitize pancreatic cancer cells to gemcitabine *in vitro*[50]. It was published that it inhibits proliferation and induce apoptosis in LNCaP prostate cancer cells *in vivo*[51]. Curcumin inhibits dose-dependent the SSC4-oral cancer cells, LoVo- human colorectal cancer cells, K1- papillary thyroid cancer cells, KKU100, KKU-M156, KKU-M213 - human biliary cancer cells [52–55]. Regarding melanoma, curcumin was reported as an antiproliferative agent on high metastatic B16F10 murinic melanoma cell line by targeting nucleotide phosphodiesterase 1A [56]. Furthermore curcumin was found to induce G2/M cell cycle arrest in case of human melanoma cells [57].

Ginger extract and 6-gingerol were found to inhibit the proliferation of rat colonic adenocarcinoma [58]. Moreover, it was proved that 10-shogaol, an important pharmacological compound from *Zingiber officinale* has the ability to promote growth of normal human skin cells [59]. Additionally literature reports G0/G1 arrest and apoptosis induced by ginger extract in case of HCT 116 and HT 29 colon cancer cell lines [60]. Chemopreventive efficacy of ginger extract was also described against hepatoma HepG2 and HLE cell lines [22]. Furthermore, aqueous extract of ginger was proclaimed to present antiproliferative activity in case of human non-small lung epithelium cancer (A549) cells and human cervical epithelial carcinoma (HeLa) [61].

The group of Lea *et al.*, published that ginger extract is active on some cancer cell lines like ovary -OVCAR and leukemia - K562, but not significantly active in case of other cancer cell lines like breast-MCF7, colon HT29, lung NCI460, prostate PCO3 and melanoma UACC62 [58, 62].

Due to the fact that apoptosis is in balance with proliferation, we have also investigated a possible proapoptotic effect of selected extracts. DAPI staining was useful in order to observe first insights of apoptosis, but real quantification of both early and late apoptotic cells has been done by double staining Annexin-FITC-7AAD. As explained in the results section these assays revealed that at a dose of 100 μg/ml the extracts present proapoptotic capacity.

Curcuma aqueous extract was proclaimed with a proapoptotic activity against human colon carcinoma LS-174-T cells [63]. Turmerone, extracted by supercritical carbon dioxide was noted to induce apoptosis in hepatocellular carcinoma HepG2 cells [46]. The group of Ozaki *et al.*, demonstrated the role of curcumin in the induction of rabbit osteoclast apoptosis along with inhibition of bone resorption [64]. Curcumin was previously reported for its proapoptotic activity in human lung carcinoma A549 cells, NPC-TW 076, human nasopharyngeal carcinoma cells, human colon cancer colo 205 cells, murine myelomonocytic leukemia WEHI-3 cells, leukaemic Jurkat cells [63, 65–67]. Curcumin was described as a proapoptotic agent against different human melanoma cells [57, 68].

On the other hand, ginger extract was reported to trigger apoptosis in case of HCT 116 and HT 29 colon cancer cell lines [60]. Zerumbone, an active agent from *Zingiber aromaticum* was describd as a dose-dependent proapoptotic agent in case of HT-29, CaCo-2, and MCF-7 cancer cells [69]. Ethanolic extract of *Zingiber officinale* triggered apoptosis in case of HepG_2_ - human hepatoma cell line [70]. [6] -paradol, a minor constituent of ginger and other related derivates were demonstrated to induce apoptosis in case of oral squamous carcinoma cell line KB and human promyelocytic leukemia (HL-60) cells [71, 72]. In addition, [6]-gingerol, the main pharmacologically active principle from ginger root was reported to induce apoptosis in case of SCC-25 oral cavity cancer cell line, different human colorectal cancer cells, human cervical cancer HeLa cells and human promyelocytic leukemia (HL-60) cells [72–75]. Furthermore, terpenoids present in steam distilled extract of ginger had the capacity to induce apoptosis in endometrial cancer cells via p53 activation [22]. [6]-gingerol was reported to inhibit melanogenesis in B16F10 melanoma cells [76].

## Conclusions

The present study can be considered of practical interest since our results supplement the data existing in the literature with new information regarding the antiproliferative, respectively proapoptotic effect of the selected extracts on murine melanoma B164A5 cell line. To the best of our knowledge this report was never done before. It is obvious that *Curcuma rhizome* ethanolic extract presented an increased effect in comparison with *Zingiber rhizome* ethanolic extract on B164A5 murine melanoma cell line regarding both proliferation and apoptosis. The increased anticancer activity may be correlated with the higher amount of polyphenols, respectively increased antioxidant capacity as detected by Folin Ciocalteu and DPPH assay. These preliminary findings are of great interest and further studies will be developed in order to characterize the extract and find pure active phytoconstituents that in a proper dose may exercise an increased antimelanoma activity. As a clear conclusion presented results indicate that *Curcuma rhizome,* a main representant of *Zingiberaceae* family may be a promising natural source for active compounds against malignant melanoma.

## Methods

### Vegetal extracts

*Curcuma rhizome* (*Curcuma longa* Linnaeus) and *Zingiber rhizome* (*Zingiber officinale* Roscoe) were achieved from University of Agricultural Sciences and Veterinary Medicine, Timisoara, Romania, Department of Plant Culture. Fresh rhizomes of both turmeric and ginger were cleaned, washed with deionised water, sliced and dried in the sun for one week, and dried again at 50°C in a drying stove for 6 hours. Dried rhizomes were cut in small pieces, and powdered by electronic mill. Extraction was performed by using ethanol as a solvent, solid: liquid ratio 1:50 at 70°C for 2 h [38].

The formal identification of the plant material was done by the specialist in the field Dr. Senior Lecturer Danciu Corina, Department of Pharmacognosy, Faculty of Pharmacy, University of Medicine and Pharmacy Victor Babes, Timisoara.A voucher specimen of this material has been deposited in a herbarium, available at UMFT Victor Babes, Timisoara-Department of Pharmacognosy, No. Ph-28.17.

### Total polyphenolic content

Total polyphenolic content of selected vegetal products was determined by Folin Ciocalteu assay. The method is based on the measurement of optical density of a primer extract which by complexation with the Folin-Ciocalteu reagent absorbs in the visible domain at λ = 720 nm (multidetection Biotec spectofotometer, UV–VIS 190–900 nm). The necessary reagents are: bidistilled water, Folin Ciocalteu reagent (Merck, Romania), Na_2_CO_3_ (sodium bicarbonate) and ethanol (Sigma Aldrich, Romania). The assay was conducted on a microplate with 24 wells and the following quantities of reagents were used: 23 μl sample; 115 μl Folin Ciocalteu reagent; 345 μl Na_2_CO_3_ (7.5%) and 1.817 ml bidistilled water. Standard curve was done using different concentrations of gallic acid (mg/ml) as standard. Absorption at 765 nm was measured. Total phenol contents were expressed in gallic acid (Sigma Aldrich, Germany) equivalents (mg gallic acid/g dry weight - DW). All determinations were performed in triplicate.

### Antioxidant capacity (DPPH assay)

This assay is based on the measurement of the reducing ability of antioxidants toward DPPH (2, 2′ diphenyl-1-picrylhydrazyl) radical. The DPPH radical-scavenging activity was determined using the method proposed by Brand-Williams *et al.*[77]. The assay was conducted on a microplate with 24 wells. DPPH (80 μM) was dissolved in pure ethanol (98%). The radical stock solution was prepared fresh. The mixture was shaken vigorously and allowed to stand at room temperature in the dark for 10 min. 200 μl of sample with 1.4 ml radical solution were added to each microplate well. The decrease in absorbance of the resulting solution was monitored at 515 nm for 30 min. The results were corrected for dilution and expressed in μM Trolox ((S)-(−)-6-hydroxy-2,5,7,8-tetramethylchroman-2- carboxylic acid) (Sigma Aldrich, Germany) per 100 g dry weight (DW). All determinations were performed in triplicate.

### B164A5 melanoma cells

B164A5 cells were acquired from Sigma Aldrich (ECACC, origin Japan stored UK). The complete growth medium (culture medium) for this cells is DMEM (Dulbecco’s Modified Eagle’s Medium), supplemented with 10% FCS (Fetal Calf Serum), 1% Penicillin/Streptomycin mixture (Pen/Strep, 10.000 IU/ml) and 2% HEPES (4-(2-hydroxyethyl)-1-piperazineethanesulfonic acid). The cells were cultured by incubation at 37°C in 5% CO_2_ atmosphere. At a confluence of 70-80% (every two or three days) the cells were passed using 0.25% Trypsin - 1 mM EDTA solution followed by centrifugation (5 minutes, 1200 rpm) and replated in T75 culture flasks at a subcultivation ratio of 1:10 to ensure optimal proliferation.

### MTT proliferation assay

MTT kit was acquired from Roche, Germany. 100 μl cell suspension containing 6×10^3^ B164A5 melanoma cells were seeded onto a 96-well microplate and attached to the bottom of the well overnight. Afterwards 100 μl of new medium containing 10% FCS and 100 μg/ml of plant ethanol extracts were added. After 48 h of incubation, 10 μl of MTT reagent from a stock solution of 5 mg/ml were added. The intact mitochondrial reductase converted and precipitated MTT as purple crystals during a 4 h contact period. After four hours the precipitated crystals were dissolved in 100 μl of solubilisation solution. Finally, the reduced MTT was spectrophotometrically analyzed at 570 nm, using a reference of 656 nm using an ELISA reader. Inhibition index was calculated as 1-absorbance sample X/absorbance sample blank.

### DAPI (4, 6-Diamidino-2-phenylindole) staining

B16 cells were seeded at a concentration of 5×10^4^ in a chamber slide system formed of 8 well glass slides in culture medium. After 24 h cells were incubated in medium containing 10%FCS and 100 μg/ml of plant ethanol extracts. The total volume added in chamber was 400 μl. Cells were incubated for 48 h and afterwards the medium was removed. Cells were washed with PBS and afterwards 400 μl of staining solution were added in each well. The staining solution consisted of a mixture of methanol and DAPI (Roche) as follows: 1 ml methanol: 2 μl DAPI (from a stock solution of 1 mg/ml). Cells were incubated for 5 minutes with this staining solution and afterwards washed with PBS and analyzed by fluorescence microscopy.

### Annexin-FITC-7AAD double staining

Cells were cultivated in a 6 well plates at a density of 80% using normal medium. After 24 h medium containing 10% FCS and 100 μg/ml of plant ethanol extracts were added. After 48 h cells were detached using trypsin, washed with ice cold PBS and resuspended in 500 μl Annexin binding buffer (1.19 g HEPES NaOH pH =7.4; 4.09 g NaCl; 0.138 g CaCl_2_ in 50 ml distillated water and diluted 1:10) at a concentration of 1×10^6^ cells/ml. Cells were centrifugate 5 min at 1200 rpm, the supernatant was discarded and the cells were resuspended in 70 μl of Annexin binding buffer. 5 μl Annexin V-FITC (ImmunoTools) and 5 μl 7AAD (ImmunoTools) were added and cells were incubated 15 min on ice and in the dark. Samples were measured by FACS on FL1 and FL3 fluorescence channels using a BD Canto II FACS DIVA device. Untreated cells were used as negative control and cells treated with 500 nM staurosporine (LC laboratories) for 24 h were used for the compensations. Flow Jo soft (7.6.3) was used for data analysis.

### Statistics

Unpaired Student t test or Two-Way ANOVA followed by Bonferroni post test were used to determine the statistical difference between various experimental and control groups.*, ** and *** indicate p < 0.05, p < 0.01 and p < 0.001 compared to control group. Results are presented as mean ± standard deviation (SD).

## Abbreviations

DW: Dry weight
DPPH: 2: 2′ diphenyl-1-picrylhydrazyl
DMEM: Dulbecco’s Modified Eagle’s Medium
FCS: Fetal Calf Serum
HEPES: 4-(2-hydroxyethyl)-1-piperazineethanesulfonic acid
DAPI: 4, 6-Diamidino-2-phenylindole
PBS: Phosphate buffered saline
GAE: Gallic acid equivalents
B164A5: Murine melanoma cells.
